# Local data commons: the sleeping beauty in the community of data commons

**DOI:** 10.1186/s12859-022-04922-5

**Published:** 2022-09-23

**Authors:** Jong Cheol Jeong, Isaac Hands, Jill M. Kolesar, Mahadev Rao, Bront Davis, York Dobyns, Joseph Hurt-Mueller, Justin Levens, Jenny Gregory, John Williams, Lisa Witt, Eun Mi Kim, Carlee Burton, Amir A. Elbiheary, Mingguang Chang, Eric B. Durbin

**Affiliations:** 1 Division of Biomedical Informatics, College of Medicine, University of Kentucky, Lexington, KY USA; 2 Cancer Research Informatics Shared Resource Facility, Markey Cancer Center, Lexington, KY USA; 3 Kentucky Cancer Registry, Lexington, KY USA; 4 Department of Pharmacy Practice and Science, College of Pharmacy, University of Kentucky, Lexington, KY USA; 5 Department of Pharmacy Practice, Center for Translational Research, Manipal College of Pharmaceutical Sciences, Manipal Academy of Higher Education, Manipal, Karnataka India; 6 Department of Computer Science, Eastern Kentucky University, Richmond, KY USA

**Keywords:** Public data commons, Local data commons, End-to-end model, Genomic data, Clinical data, Data harmonization, Data standardization, Data integration, Cancer registry

## Abstract

**Background:**

Public Data Commons (PDC) have been highlighted in the scientific literature for their capacity to collect and harmonize big data. On the other hand, local data commons (LDC), located within an institution or organization, have been underrepresented in the scientific literature, even though they are a critical part of research infrastructure. Being closest to the sources of data, LDCs provide the ability to collect and maintain the most up-to-date, high-quality data within an organization, closest to the sources of the data. As a data provider, LDCs have many challenges in both collecting and standardizing data, moreover, as a consumer of PDC, they face problems of data harmonization stemming from the monolithic harmonization pipeline designs commonly adapted by many PDCs. Unfortunately, existing guidelines and resources for building and maintaining data commons exclusively focus on PDC and provide very little information on LDC.

**Results:**

This article focuses on four important observations. First, there are three different types of LDC service models that are defined based on their roles and requirements. These can be used as guidelines for building new LDC or enhancing the services of existing LDC. Second, the seven core services of LDC are discussed, including cohort identification and facilitation of genomic sequencing, the management of molecular reports and associated infrastructure, quality control, data harmonization, data integration, data sharing, and data access control. Third, instead of commonly developed monolithic systems, we propose a new data sharing method for data harmonization that combines both divide-and-conquer and bottom-up approaches. Finally, an end-to-end LDC implementation is introduced with real-world examples.

**Conclusions:**

Although LDCs are an optimal place to identify and address data quality issues, they have traditionally been relegated to the role of passive data provider for much larger PDC. Indeed, many LDCs limit their functions to only conducting routine data storage and transmission tasks due to a lack of information on how to design, develop, and improve their services using limited resources. We hope that this work will be the first small step in raising awareness among the LDCs of their expanded utility and to publicize to a wider audience the importance of LDC.

## Background

Public Data Commons (PDC) provide interoperable services for hosting data and computing infrastructure including software tools and applications for managing, analyzing and sharing data in a multidisciplinary research community while administrating data governance and security [1].

The National Cancer Institute (NCI) Cancer Research Data Commons (CRDC) covers cloud-based data science infrastructure for multiple NCI DCs that provide secure access to a large, comprehensive, and expanding collection of cancer research data [2]. Five DCs exist under the NCI CRDC: (1) Genomic Data Commons (GDC) [3] collects, processes, and analyzes data on a project-level basis and provides uniformly processed genomic and associated clinical data using GDC Data Harmonization [4] and GDC Data Dictionary [5]. (2) Imaging Data Commons (IDC) [6] provides cancer imaging data, computational resources, and big data analysis tools through the Google Cloud Platform and Imaging Data Portal [7]. (3) Integrated Canine Data Commons (ICDC) [8] provides a harmonized and standardized public resource for exploring, analyzing, and understanding the biological relationships between human and canine cancers by utilizing the ICDC data model and harmonization process. ICDC users can access and analyze data through the Seven Bridges Cancer Genomic Cloud (SBCGC) [9]. (4) Proteomic Data Commons [10] helps to understand the risk, diagnosis, development, progression, and treatment of cancer by providing highly curated and PDC standardized [11] biospecimen, clinical, and proteomic data with analysis tools and cloud resources. (5) Clinical Trial Data Commons (CTDC) [12] is being developed to provide clinical trial data that will help researchers understand the relationship between tumor molecular characterization, treatment, response and progression. Data is harmonized in the CTDC, standardized by the CTDC data model, and made available through the SBCGC. Data access control and security is handled through the database of Genotypes and Phenotypes (dbGaP), providing two levels of access for the large-scale genetic and phenotypic datasets, namely, publicly accessible high level summary data and authorized access to individual and raw data [13].

Collaboration across continental boundaries has also led to the establishment of international PDCs. For example, the Human Cell Atlas (HCA) Data Portal hosts multi-omic open data generated by the international scientific community and processed by standardized pipelines utilizing WDL Analysis Research Pipelines to comprehensively characterize human cell types and states [14]. The International Cancer Genome Consortium Accelerating Research in Genomic Oncology (ICGC ARGO) Data Platform [15] provides clinical and genomic data donated from 13 countries, harmonized [16] and standardized [17] by the ICGC Data Coordination Center (DCC) Bioinformatics team [18]. International Agency for Research on Cancer (IARC) [19] is the specialized cancer agency of the World Health Organization and promotes international collaboration in cancer research as collaborating with large number of institutions from 141 countries.

In addition to these PDC examples, large community-based data repositories are also actively used. For example, Gene Expression Omnibus (GEO) [20] hosts a publicly available functional genomics data from microarray and NGS sources, and provides tools to query and download data. All of Us Research Hub [21] stores health data from more than 316,000 participants with various medical concepts from across the United States and standardized with the Observational Medical Outcomes Partnership (OMOP) Common Data Model (CDM) [22].

Treehouse Genomics [23] shares clinical and genomic data obtained from more than 12,000 child tumor samples processed with an RNA-Seq pipeline developed by the UC Santa Cruz Computational Genomics Lab. The Blood Profiling Atlas in Cancer (BloodPAC) consists of members from multiple organizations and institutes and facilitates the exchange of raw and processed data generated by the liquid biopsy research community. The Oncology Research Information Exchange Network (ORIEN) [24] is an alliance of cancer centers and provides M2Gen’s harmonized and standardized information to cancer centers, biopharmaceutical companies, researchers, and scientists.

As sequencing and molecular characterization technology has evolved, the volume of data included in these PDCs has grown dramatically over the last 10 years [25, 26], and the data shared by these PDCs are actively used for biomedical and healthcare informatics research [27, 28]. While the creation and use of PDC has been extensively documented, institutional and organization-based local data commons (LDC) have received relatively little attention. This lack of attention on LDC is unfortunate, considering their importance in data curation and sharing. Within an organization, LDCs are uniquely positioned closest to their data sources to capture up-to-date and detailed patient information, specimen data, and sequencing results as soon as the data is generated. In contrast to their importance, however, there is a lack of documentation and guidance for resourcing, building, and maintaining LDC. Moreover, the sparse documentation [29–34] does exist typically does not provide practical advice on how to get started with planning, data modeling, and implementing a new LDC within an organization.

To begin to address these deficiencies, we describe the relationships between LDC and other DC, including PDC, with three different deployment models. To simplify the definition of these models, we will consider use cases where an LDC contains only clinical and genomic data.

**Fig. 1:**
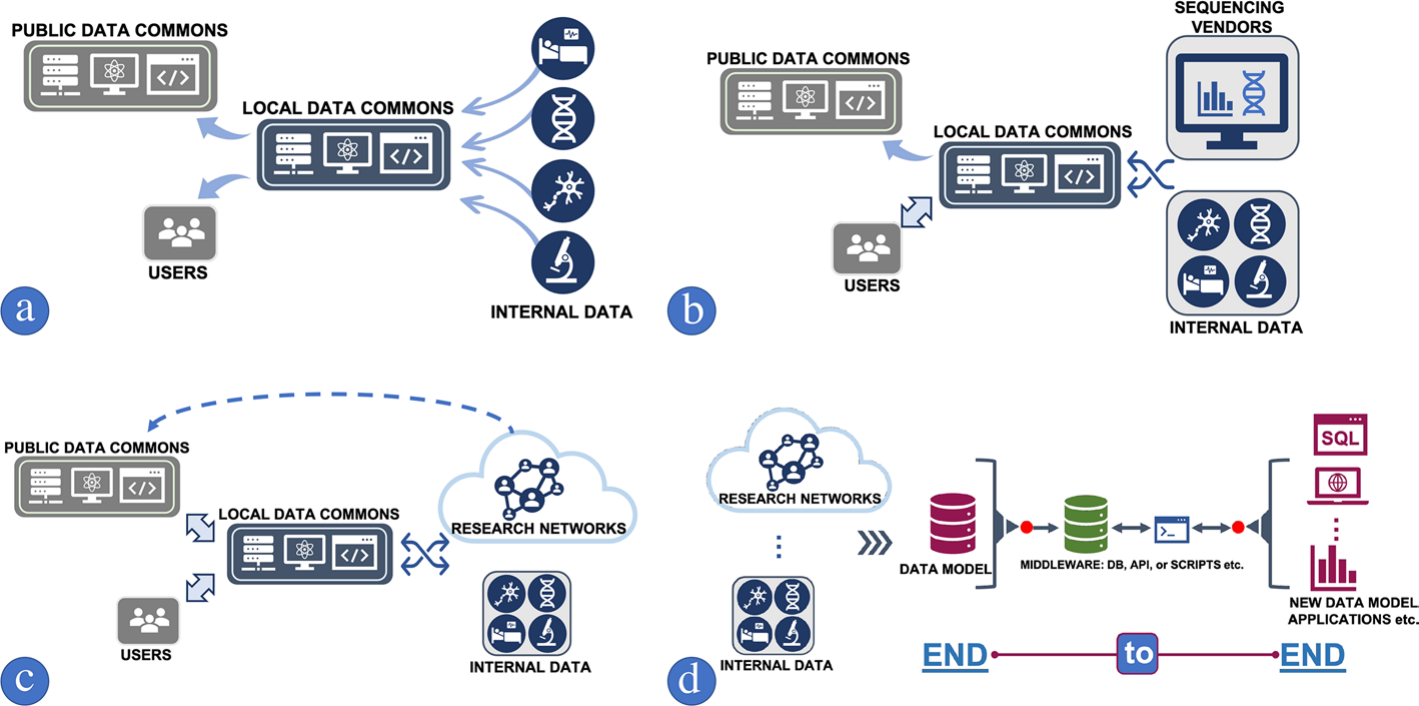
Three types of Local Data Commons (LDC) service models: **a** Liaison model; **b** Enterprise model; **c** Network model; **d** End-to-end data sharing model

*Liaison model*: This LDC is a liaison of data as shown in Fig. 1a. In this model, the LDC may implement very few of the FAIR principles [35] (Findability, Accessibility, Interoperability and Reusability). Although the LDC primary function is to collect and distribute data, it has to strictly follow heterogenous data governance guidelines for each project and needs to perform data management tasks including processing patient consent forms, reformatting data based on a requested data model, managing data use agreements, shipping and tracing specimens, and maintaining sequencing results, etc. Therefore, even this simple model will incur significant costs in terms of labor, time, and system resources.

*Enterprise model*: This LDC supports local users only, as shown in Fig. 1b. LDC utilizing this model will have another layer of services including data analysis, harmonization, and computing resources. Compared to the *Liaison Model*, these enterprise DCs are actively engaged in data transfer with community research networks or sequencing vendors to collect, deliver, standardize, and harmonize data to provide services for internal researchers and project investigators. Since different sequencing vendors have different data processing pipelines, reporting systems, and data models, these DCs require a professional team consisting of system and security administrators, database analysts, data scientists, and biomedical informatics specialists. Since these DCs are typically implemented with a minimal amount of staffing and system resources, data harmonization and standardization are a significant challenge compared to a PDC.

*Network model*: This LDC is a hybrid PDC, serving as both a data provider and an active consumer of data from a PDC. Certain data in this LDC model cannot be shared with the PDC due to data governance issues, incomplete data from ongoing projects, confidentiality, or intellectual property concerns [36–38]. A primary characteristic of this type of LDC is that the data from PDC is used to validate and integrate its own internal data. Ironically, this LDC needs to standardize and harmonize the already harmonized PDC data again for local integration and analysis, since it will be comparing results from the PDC against its own local data sources. This local re-harmonization of data can be problematic since different PDCs use different harmonization pipelines. In fact, there are hundreds of informatics tools [39–44] involved in building PDC data pipelines; therefore, re-harmonizing the data locally may be less efficient than simply integrating the original source data, if it can be obtained. Moreover, re-harmonized data from a PDC genomic pipeline may not reproduce the original genomic variant reports, particularly when custom, unpublished assays and proprietary databases were used to generate the reports. As a consequence, re-harmonized data and the original PDC data cannot be compared directly, sometimes causing confusion among investigators. In Fig. 1c, the network model LDC interacts with research networks and the PDC as both a data provider and a consumer, compared to Fig. 1b, the enterprise model where the LDC only has the role of facilitating data transfer.

Although considerable effort has been made to harmonize and standardize data in PDCs, their differing harmonization pipelines and data models can inhibit data integration and comparison efforts. Indeed, the issues resulting from various harmonized data and pipelines are continuously propagated back and forth while processing reciprocal data between PDC and LDC. LDCs struggle to reconcile internal data harmonization with externally harmonized data from PDC, and there are few practical resources and guidelines to help with this problem. In most cases, LDCs are forced to find solutions in isolation and often perform redundant work across organizations due to the inherently limited public communication among LDC administrators.

The Kentucky Cancer Registry Cancer Research Data Commons (KCR CRDC) is a DC housed at one of the leading Surveillance, Epidemiology, and End Results (SEER) cancer registries that supports collection and integration of genomic information with cancer registry records [45, 46]. The KCR CRDC has been actively engaged in the cancer informatics research community, handling genomic reports from multiple sequencing vendors, and closely working with the University of Kentucky Markey Cancer Center (MCC) to provide data harmonization services for multi-disciplinary research; thus, categorized as a *Network Model* LDC. The KCR CRDC has integrated and linked population-based registry data from the Kentucky SEER registry along with more than 4,000 cases of tumor-specific genomic data. Linked data includes demographic, clinical, treatment and outcome data, electronic pathology data, and additional abstracted data captured by the MCC’s Molecular Tumor Board (MTB).

The Kentucky Cancer Registry faced numerous challenges while establishing the CRDC due to the rapid evolution of DC methods, along with the ongoing maturation of technologies for producing, analyzing, and reporting data.

As noted earlier, different PDCs employ different data models and harmonization pipelines, and therefore, a “one size fits all” LDC model is not adequate. Even within a single organization, expectations and required technologies for developing an LDC will vary among different fields of specialization. Therefore, the LDC will need to employ varying workflow solutions and data processing pipelines while operating with limited resources.

Lessons learned from our experience may be helpful for other DC under development that plan to integrate genomic data in all of its forms. Our proposed method can inform decisions to build a new DC within an organization by providing guidance on the function of the DC, the data preparation necessary to successfully populate the DC, and how to serve the needs of the local research community. For those organizations that already have a LDC, this article can help to improve data maintenance, data quality, data sharing, and provide advice on how to extend and enhance services for investigators.

## Results

### cBioPortal as an instance of end-to-end staging database model

**Fig. 2:**
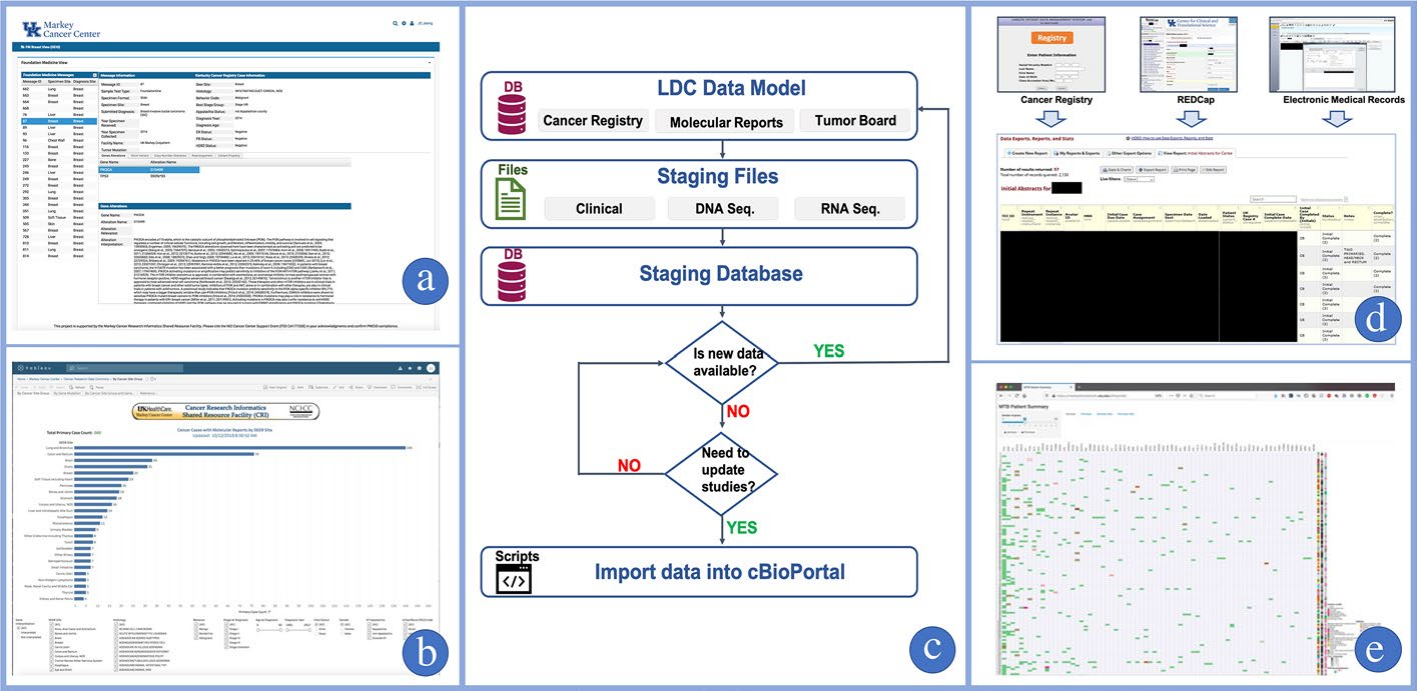
Applications and workflow: **a** Online molecular report explorer utilizing LabKey. **b** Online statistics tool for molecular report utilizing Tableau. **c** MCC cBioPortal management system utilizing end-to-end model. **d** Data linkage application gathering all information in one view and committing linkage with one click. Identifiable patient information is masked with a black box. **e** MCC Molecular Tumor Board on-demand data visualization tool

MCC cBioPortal is one of the applications using staging databases in the proposed end-to-end model as shown in Fig. 1d. It is important to note that cBioPortal [47] was originally designed to host results from completed studies. Therefore, ongoing studies are more difficult to maintain in cBioPortal due to the absence of tools for updating existing records. In addition, the required data elements and formats are not matched with KCR CRDC’s data model. To address this issue, cBioPortal provides an interface via so-called staging files, which enable data to be imported into cBioPortal. Once the files are created, provided Python scripts read the files, calculate basic statistics for visualization, and insert the corresponding values into cBioPortal’s DB. Due to the required preprocessing steps, inserting values directly into cBioPortal DB without using the provided staging files and scripts is very difficult and not recommended, especially when the sustainability of services is considered. Though creating staging files is a time-consuming but necessary process, and it can be semi-automated using the proposed end-to-end staging database model. For example, the Markey cBioPortal (cbioportal.kcr.uky.edu) server uses Python scripts to pull data from a MySQL database where KCR data and genomic reports are consolidated. Once the data is retrieved, it is used to create staging files based on the staging file format guidelines specified in the cBioPortal documentation (docs.cbioportal.org). When updating ongoing studies, it is useful to create staging databases with the required data model rather than on-demand updates. With this approach, whenever study cases are added or updated, the staging database searches and compares the existing data model and updates the corresponding data in the staging database as depicted as a decision process in Fig. 2c. Updating existing studies in cBioPortal requires much higher computing power and time than the deletion and insertion process. MCC cBioPortal works around this issue by completely rebuilding cBioPortal’s database from the scratch for every release. As a result, a wholesale redeployment is much faster than updating individual studies. Figure 2c shows overall workflow to maintain MCC cBioPortal where the small icons in each stage indicate data type: database (DB), file type (files), and programming codes (scripts).

### Data linkage application

Although genomic data annotated with rich clinical data is critical for designing and validating many types of studies, collecting and integrating this data while maintaining high accuracy is challenging and time consuming. The key to maintaining the KCR CRDC is creating and maintaining high-quality linkages between genomic data and population based KCR data. Genomic data may lack clear identifiers needed to link to the patient, case, and specimen, therefore human curation is essential for achieving highly accurate linkages. To help these curation tasks, the KCR CRDC has developed a human-interactive data linkage application utilizing the LabKey application server where multiple data sources such as pathology reports and correlative data in relational databases can be searched. With this simple linkage and search tool, project investigators are able to search and display all resources on one screen while performing one-click linkages through a user-friendly graphical user interface (GUI). Figure 2d shows one of the applications that KCR CRDC developed and used for performing data linkages. The application displays all relevant information on a screen by pulling data from the cancer registry, electronic medical records, and other supporting data collected from multiple facilities with REDCap. Due to the confidentiality of patient data, identifiable information has been masked in the figure.

### Secure genomic reporting tools

Delivering a large cohort of genomic reports along with structured supporting data files may be problematic due to large data volumes. In addition, the researchers requesting the data may have little or no experience with handling large, sensitive data sets. To alleviate this problem, the KCR CRDC has developed user-friendly, secure, access-controlled genomic reporting tools in LabKey [48] and Tableau with the capacity to search and filter data as shown in Fig. 2a, b respectively. Accessing these services is limited to authorized VPN connections with read only permissions. Also, since the tools rely on the end-to-end design model as shown in Fig. 1d, any user requests, including data integration and visualization, can be easily accomplished without modifying or changing the original data model, per-task data access policies, or consuming large amount of computing resources.

### On-demand data visualization

The MCC Molecular Tumor Board (MTB), part of the University of Kentucky HealthCare enterprise, is a statewide service forum for expert clinicians, pathologists and scientists to discuss and analyze tumor genotypes and molecular abnormalities in order to recommend patient-specific, targeted therapies [49]. The KCR CRDC supports the MTB by maintaining and integrating MTB-reviewed patient data and developing applications for the retrieval and visualization of patient data. The MTB data abstraction tool (https://markeybiostattools.uky.edu/) utilizes data from OnCore and the KCR DB while taking advantage of a self-hosted R-Shiny server to provide a rich visualization front-end. This tool is specifically designed for custom requests that are not easily fulfilled by existing applications. As an additional layer of security, data are de-identified and stored independently from any linkage to their original database. Figure 2e shows that the information from MTB patients can be visualized with mutated genes and types, SEER cancer sites, treatments, and recommendations. To protect patient confidentiality, the service is restricted to authorized users only.

## Discussion

High-throughput technologies have been actively used in the study of genomics and proteomics, including genetic variants, gene and protein expression, and epigenetic modifications [50–54]. In addition, genome and exome sequencing have shown great potential for aiding in cancer diagnosis, precision treatment, and drug development, and have been actively used in multidisciplinary research areas, such as community-based research programs [55–57].

Furthermore, emerging new technologies like CRISPR and single cell sequencing have increased the resolution of NGS and improved the accuracy of results [58, 59]. Because of its popularity and the increasing demand for NGS, huge volumes of data have been generated as well as a wide variety of analysis platforms and data governance frameworks [60–63]. This data and complexity explosion has also brought unprecedented challenges to data commons in terms of data governance and subsequent costs for data collection and harmonization [64, 65]. To overcome these challenges and improve data accessibility and usability, significant effort has been made to build PDC, and indeed, many PDCs have been serving research requests with their own standardized and harmonized data and have been widely cited in the literature. Ironically, certain data in PDCs may not be as useful as data hosted in LDC due to the lack of services, such as failing to provide timeliness of the data, inconsistent and non-standard clinical annotations, extensions to data elements, and data quality validation. Nonetheless, even LDCs that existed before PDC became widespread have not gained adequate public attention, and as a result, have lacked documentation and guidance on creation, administration, and maintenance. As a result, a LDC can be vulnerable to technology changes that make them difficult to discover, access, and update.

Like other LDCs, KCR began with the Liaison model that flowed naturally from its unique role in collecting data from hospitals and consolidating the data into the SEER registry. As the data in the KY SEER registry grew in complexity and volume, researchers began requesting access to this valuable, curated data source, making it popular among the MCC, national, and international research community. At this stage, the LDC became actively involved in research projects, and data were exposed to a variety of research groups, which resulted in a need to harmonize and integrate additional data sources that were linked to the KY SEER registry; thus, implementing security procedures and data sharing polices were necessary along with developing data protection and processing pipelines to make the data accessible in a secure manner. Since data from a single institution could be biased and have small sample sizes, the network model was employed to aggregate similar data sources from other institutions to augment the KY LDC and further build an advanced research data source. In addition, as the size of the network grew, an end-to-end data sharing model was required to accomplish requests from multiple organizations for data preparation and data transfer methods that conformed to a variety of other institutional data models and security guidelines. These changes all needed to be made while minimizing manual human intervention due to constrained resources at the KCR. Indeed, handling this data expansion in a secure manner without exhausting all available resources is one of the critical issues continuing to face KCR, especially while the complexity of collaboration networks continues to expand. The KCR CRDC utilized a standard vocabulary for clinical data as specified by North American Association of Central Cancer Registries (NAACCR), used by all cancer registries throughout North America and Canada. However, we recognize the value of data interoperability among LDCs. Several initiatives are underway to map the NAACCR data model to the OMOP Common Data Model which will address this limitation. As LDCs expand their dataset with new elements, we recommend choosing standard vocabularies already defined within the OMOP model when possible. The KCR is continuously working on implementing new models that can optimize the cost of effort, sustainability of the data model, efficiency of data sharing, and the compatibility of applications for discovery, search, and output of the data. It is clear that the amount of data and collaborations will continue to grow along with the importance of this data resource as shown by growing world-wide research collaboration efforts such as IARC and recent efforts to study the COVID-19 pandemic. The KCR CRDC is supporting an international collaboration between the MCC and Manipal Academy of Higher Education (MAHE). As part of this initiative, sequencing and clinical data generated by MAHE will be incorporated into the KCR CRDC, which will also be made available to researchers at MAHE and MCC. The bottom line is that the role of LDC should not be neglected in data commons community. Although the focus of this paper is on integrating cancer registry and genomic data, the end-to-end data sharing model we describe is flexible enough to apply to many other biomedical data sources without extensive modification. For example, ORIEN projects involve the identification of patient cohorts and acquisition and shipment of specimens for sequencing, and the download and transfer of WES data to collaborating investigators. For these projects, the KCR CRDC is responsible for providing patients and specimen data based on M2Gen’s data model. To accomplish this, the KCR CRDC collects and integrates required information from multiple SRFs using the end-to-end data model by utilizing REDCap for specialized data capture and custom scripts for harmonizing the data, instead of changing the SRF's own internal data model. It is beyond the scope of this article to provide approaches and methods to address all requirements and activities of an LDC, but we hope that this work lays out some guidelines delineating the essential roles, activities, and potential of LDC and explains what services are expected and resources required as institutions consider the development or expansion of their own LDC.

## Conclusions

Although LDCs commonly have been relegated to the role of passive data providers for PDCs, there remains an enormous potential for LDCs to improve institutional data quality and deliver high value for translational research. The lack of clear documentation on how to design, develop, and create services limits many LDCs to conducting only routine processes. It is time to wake up the sleeping potential of LDC, and we intend this work to be a small step toward helping the LDC community by promoting active data sharing with enhanced data quality and coverage, working in conjunction with PDCs in the community of data commons.

## Methods

The fact that vastly differing pipelines and data models among PDCs suggests that monolithic systems [66] processing all data into a single pipeline is not an ideal choice for the LDC. Instead, a flexible end-to-end model as shown in Fig. 1d may better fit the roles of LDC accommodating a wide range of scientific needs and various tasks such as the shipping and tracking of specimens, data transfer and allocation, and data harmonization and access.

**Fig. 3:**
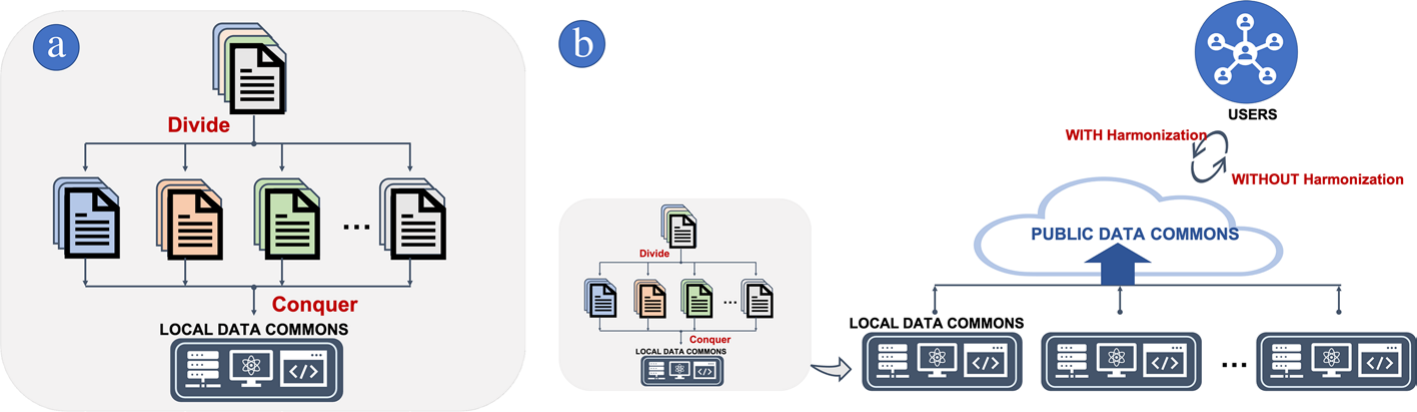
Divide-and-conquer and bottom-up data intergration method: **a** Sequencing data are divided based on sequencing vendors, assay types, and research types, etc. and then data are integrated into LDC. **b** LDC’s data produced by divide-and-conquer approach are submitted to PDC (bottom-up). Users can have choices to select original or harmonized data. Note that LDC can be both a consumer and data provider of PDC

An alternative way to harmonize the data in LDC is to use a divide-and-conquer and bottom-up approach. At the divide-and-conquer stage, sequencing results from the same vendor or research network are grouped together and then standardized and harmonized within a corresponding group as shown in Fig. 3a. As shown in Fig. 3b, in the bottom-up approach, an individual LDC submits the data processed by a divide-and-conquer approach to PDC which then integrate the original and harmonized data. For the data provider this approach reduces costs and effort when preparing data submissions. As a data consumer, LDC may simply integrate PDC data without running re-harmonization pipelines that require expensive computing resources, data storage, and staff resources. As a result, LDC can focus more on improving the service features, performance, and output.

**Fig. 4:**
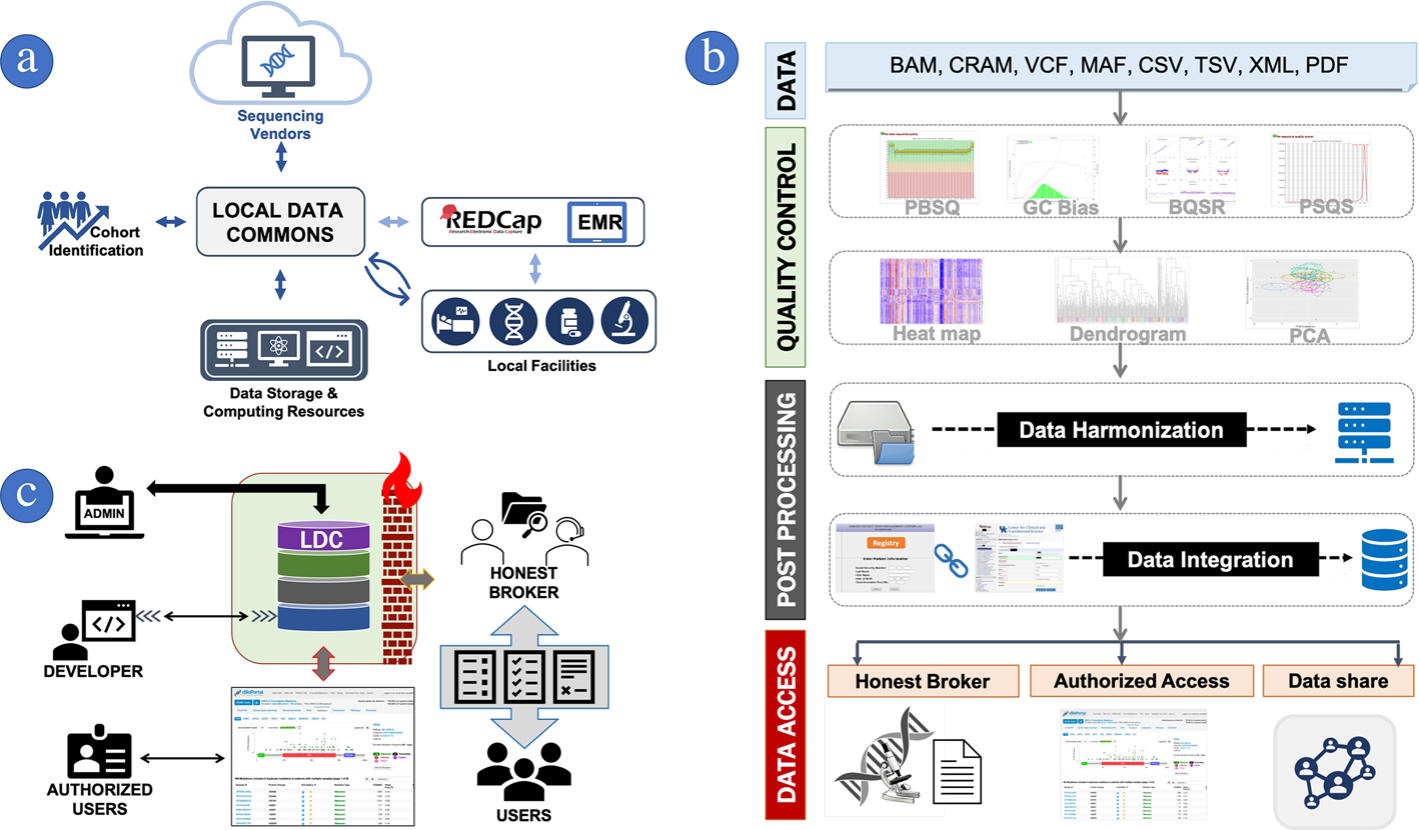
LDC's core services and workflow: **a** LDC can facilitate molecular data processing by orchestrating core services and interacting with other facilities. LDC can help data collection by utilizing REDCap or EMR applications. **b** Workflow for processing molecular data. **c** Role-based access control and user services for data access

We identify seven key LDC core services, with corresponding workflows shown in Fig. 4: (1) cohort identification and genomic sequencing facilitation, (2) management of molecular reports and infrastructures, (3) quality control, (4) data harmonization, (5) data integration, (6) data sharing, and (7) data access control.

*Cohort identification and genomic sequencing facilitation*: Genome sequencing (GS) data can generally be categorized into one of two types, clinical or research, based on intended purpose, which informs how the data are managed, processed, and disseminated. In addition, a DC may need to work with both internal and external collaborators, with implications for how data access is controlled and audited. For clinical use, LDC administrators should establish and maintain communication channels with the laboratory and sequencing facilities. Communication is crucial for maintaining quality control and data management tasks, such as correcting and clarifying specimen collection dates, sequencing dates, details of the sequencing platform, assay characteristics, and sequencing library kits. Information about the personnel in charge of preparing specimens and sequencing libraries will also be beneficial for monitoring batch effects, which are discussed later in this paper. When GS data are generated for research purposes, the role of a LDC is to identify study cohorts, facilitate data transfer, and standardize linked data into common data models and various data formats. During data preparation in the LDC, some data elements are not always available and will need to be collected and linked from other internal sources. In this scenario, the LDC may use REDCap [67] for manual data collection or abstraction.

To improve the efficiency of data collection, a LDC may load any existing data values into a REDCap form, requiring the entry of only missing data elements, thus reducing overall data abstraction efforts. Figure 4a shows that the LDC may either directly communicate with local facilities or use REDCap or another application to collect data. In order to secure GS data and reduce the likelihood of accidental breach of confidentiality, it is critical that the LDC create and maintain anonymous identifiers for the data, including the origin of the facility, the individual patient, any linked case-level data, and specimen information. These identifiers can also help expedite communication among facilities since they can be more safely shared over regular communication channels such as e-mail.

The most essential patient, case, and specimen data elements for GS requests are summarized in Table 1.

**Table 1:** Essential data elements of patients and samples

Category	Data elements
*Patient*	
Patient demographics	Name, SSN, sex, race, birth date, address, etc
Disease diagnosis	Date, site, histology, behavior, grade, stage, etc
Disease treatment	Course, date, type (surgery, radiation, chemo, etc.), agents
Long term disease outcomes	Date of last contact, vital status, recurrence status, etc
*Sample*	
Specimen ID	Unique specimen ID
Specimen Site	Blood or body tissue that is taken for medical testing
Specimen Type	Fresh frozen, FFPE, slide, etc
Date of Collection	Specimen collection date
Tumor specimen	Total tissue volume, tumor purity by stain, tumor nuclei percentage, etc

*Molecular reports and infrastructures* Depending on sequencing assays and vendors, variant reports are delivered in either industry-standard data formats (e.g., BAM, CRAM, and VCF) or vendor specific formats (e.g., XML, PDF, TXT, etc.) as depicted in Fig. 4b. If vendors provide data in nontraditional formats, the LDC should be responsible for collecting additional information, including the data dictionary, bait/target interval Browser Extensible Data (BED) files, and the reference genomes used for variant analysis. Due to the high volume of data, the complexity of analysis pipelines, and the security of patient information, Health Insurance Portability and Accountability Act (HIPAA) compliant high-performance computing (HPC) and/or cloud-based resources may be required for secure processing. Data storage requirements may vary widely depending upon the sequencing assay types. Overall data volumes may also be impacted as new sequencing vendors are added with accompanying historical sequencing results; therefore, preparing at least twice the estimated size of required file storage is a good rule of thumb.

*Quality control* Quality control (QC) as shown in Fig. 4b and file corruption tests must be conducted before accepting data into the LDC. FastQC [68] and GATK [69] provide tools to measure per-base sequence quality (PBSQ), GC bias, Base quality score recalibration (BSQR), and per-sequence quality scores (PSQS). Discrepancies between HUGO Gene Nomenclature [70] and reported gene names should be checked as well. Due to external influences such as laboratory conditions, reagent lots and personnel differences, high-throughput technologies may result in *batch effects* in which the results are biased by technical processes rather than biological or scientific factors in a study [71]. Batch effects [71–75] can be visually detected by projecting the data onto a heatmap [76] where *x* and *y*-axes are ordered by surrogates such as, sequencing date and sequencer, sequencing facility, specimen type, personnel preparing library or samples, etc. Incorporating more detailed information will improve the chance of detecting batch effects, and LDC are the optimal environment to detect batch effects. If issues are identified, the LDC may propose resequencing the samples and facilitate the data replacement. Hierarchical clustering analysis (HCA) [77] and principal component analysis (PCA) may also be applied [78] to detect batch effects.

*Data harmonization* Because genomic data are often stored in semi-structured formats and are produced from different genome builds, the raw data [69, 79–84] may need to be reprocessed for data harmonization and to generate the most up-to-date annotations. However, such an undertaking often requires a large investment in compute and storage infrastructure. For example, the KCR CRDC developed cloud computing genomic data processing pipelines, bam2vcf [84], but using this pipeline was expensive due to the high costs of moving data in and out of public cloud storage and the overhead of managing the HIPAA-compliant cloud-compute resources. Although harmonized data obtained from monolithic PDC systems where all genomic data are reprocessed by a single pipeline have consistent and seamless data elements, we propose a divide-and-conquer data harmonization approach where data are harmonized together by vendor or research network as shown in Fig. 3a.

In addition to the high cost of maintaining infrastructure for a monolithic system, there are three other reasons why a divide-and-conquer approach is more appropriate for LDCs. First, since some sequencing vendors employ their own patented technology and custom knowledge bases, conventional pipelines are unable to reproduce their original mutation reports; as a result, a reprocessing approach may lead to external cross validation issues caused by data discrepancies between the original and reprocessed data. Second, under a divide-and-conquer model, data reprocessing for the sole purpose of reference genome harmonization may not be necessary. For example, the GRCh37 reference genome was released in 2009, and now, most NGS data processing has transitioned to the newer GRCh38 reference, released in 2014. During this transitional period, integrating data into a LDC from multiple reference genomes could be a challenge because mutational reports generated with GRCh37 might not have been comparable to reports based on GRCh38. Although there are concerns about converting identified variants from different genome builds [85], LiftOver [69, 86] has been widely used in the community to cross-translate mutation reports captured by different versions of a reference genome. LiftOver does not reprocess raw data, but rather cross-maps variants between different genome builds. Since the data harmonization in a divide-and-conquer model only considers one vendor at a time, LiftOver may be a better solution than reprocessing raw data to standardize on a single reference genome in an LDC. 

Finally, at the conquer stage of the divide-and-conquer approach, data harmonization can be achieved by integrating mutations using commonly available annotation tools. Many of these tools are readily available and used by major data integration portals [47, 87]. For example, Ensemble Variant Effect Predictor (VEP) determines the effect of identified variants [88], ClinVar reports relationships among human genetic variations and phenotypes [89], gnomAD provides aggregate and harmonized disease-specific and population genetic data [90], and OncoKB annotates the biological consequences and clinical implications (therapeutic, diagnostic, and prognostic) of genetic variants in cancer [91]. Data harmonization is depicted in the third layer, POST PROCESSING in Fig. 4b, and Table 2 summarizes several essential harmonization tools for LDC.

**Table 2:** Essential tools for genomic data commons

Products	Information
*QC*	
FastQC	https://www.bioinformatics.babraham.ac.uk/projects/fastqc/
Trimmomatic	http://www.usadellab.org/cms/?page=trimmomatic
IGV	https://software.broadinstitute.org/software/igv/
SeqMonk	https://www.bioinformatics.babraham.ac.uk/projects/seqmonk/
UCSC	https://genome-store.ucsc.edu/
*Lift Over*	
UCSC LiftOver	https://genome.ucsc.edu/cgi-bin/hgLiftOver
Picard LiftOver	https://broadinstitute.github.io/picard/
Chain Files (hg38 to hg19)	https://hgdownload.soe.ucsc.edu/goldenPath/hg38/liftOver/hg38ToHg19.over.chain.gz
Chain Files (hg19 to hg38)	https://hgdownload.soe.ucsc.edu/goldenPath/hg19/liftOver/hg19ToHg38.over.chain.gz
*Annotation*	
Funcotator	ftp://ftp.broadinstitute.org/bundle/funcotator/
OncoKB	https://github.com/oncokb/oncokb-annotator
ANNOVAR	https://doc-openbio.readthedocs.io/projects/annovar
VEP	https://uswest.ensembl.org/info/docs/tools/vep/script/index.html
ClinVar	https://ftp.ncbi.nlm.nih.gov/pub/clinvar/ https://www.ncbi.nlm.nih.gov/clinvar/docs/linking/
VarScan	http://varscan.sourceforge.net/
*Data Format*	
fmi-converter	https://github.com/cBioPortal/fmi-converter
VCF2MAF	https://github.com/mskcc/vcf2maf
BAM2VCF	https://github.com/crimcc/bam2vcf
samtools	https://www.htslib.org/
bedtools	https://bedtools.readthedocs.io
vcftools	https://vcftools.github.io/index.html
bcftools	https://github.com/samtools/bcftools
*Data Resources*	
gnomAD	ftp://ftp.ensembl.org/pub ftp://ftp.broadinstitute.org/pub/ExAC_release/
Genome (hg38)	https://hgdownload.soe.ucsc.edu/goldenPath/hg38/bigZips/hg38.fa.gz https://console.cloud.google.com/storage/browser/genomics-public-data/resources/broad/hg38
Genome (hg19)	https://hgdownload.soe.ucsc.edu/goldenPath/hg19/bigZips/hg19.fa.gz
Broad Institute Data Bundle	ftp://ftp.broadinstitute.org/bundle/
UCSC Table Browser	https://genome.ucsc.edu/cgi-bin/hgTables
Sequencing Vendor Specific Data	FASTQ, SAM, BAM, CRAM, BED, XML, PDF, etc
*Data Integration*	
cBioPortal	https://github.com/cBioPortal/cbioportal https://github.com/cBioPortal/cbioportal-docker-compose
JupyterHub	https://jupyter.org/hub
Genomic Data Commons	https://github.com/NCI-GDC
GDC Data Access	https://gdc.cancer.gov/access-data/gdc-data-transfer-tool
GDC Pipelines	https://docs.gdc.cancer.gov/Data/Introduction/
Cancer Genomic Data Server	https://github.com/cBioPortal/cgdsr
cBioPortal R package	https://www.bioconductor.org/packages/release/bioc/html/cbaf.html

*Data integration* Genomic data often include only limited information focused on the active disease as shown in Table 3. Important factors such as treatment histories and other complications are often neglected although they could have significant impacts on the downstream data analysis.

**Table 3:** Basic genomic data dictionary

Report type	Data elements
*DNAseq*	
Variant Type	SNP, insertion, deletions, copy number variant, rearrangement
Mutation	Gene name, position, coding sequence effect, protein effect, allele fraction, transcript ID, strand
Copy Number Variant (CNV)	Copy Number, gene name, involved exons, position, CNV type (e.g., loss, amplification)
Rearrangement	Gene names, positions, rearrangement types (e.g., fusion, truncation, etc.)
Microsatellite-instability	Result values, category value (i.e., MSS, MSL, MSH)
Tumor Mutational Burden	Unit (e.g., Mutations per Million Base), Result values
*RNAseq*	
Expression	Gene name, expression unit (i.e., RPM/CPM, RPKM/FPKM, TPM, TMM, etc.), expression level, gene type (e.g., mRNA, lncRNA, circRNA, etc.), transcript ID
Fusion	Positions, Junction read count, fusion sequence, expression unit

The KCR CRDC aims to build a population-based genomic data commons that continuously seeks out and collects genomic data from providers across Kentucky. To maximize the data usability in the KCR CRDC, genomic data are linked to demographic, diagnostic, clinical and outcome data from the cancer registry. Since data accuracy and integrity are the most important prerequisites for the data integration, interactive human curation is necessary and recommended. In order to make successful linkages, at least two patient identifiers are needed in the source records such as first and last name, social security number (SSN), date of birth, and medical record number (MRN). Since human curation is time consuming and labor intensive, KCR CRDC developed a user-friendly application that permits the curator to easily review matched records and perform linkages with a single click, as shown in Fig. 2d. Human curation is always vulnerable to mistakes. However, when linkage errors do occur, there may be opportunities to identify them during cohort identification operations or within other layers of the data integration process, where the error may be easily corrected. The data integration is depicted in the second stage of the third layer in Fig. 4b.

*Data share* Sharing data among local DCs is a common way to obtain higher coverage of genomic data for certain disease cohorts or to collect more detailed patient data that are not available in PDC such as more current disease treatment courses and outcomes, additional clinical test results, pathology reports, specimen collection protocols, etc. There are two important prerequisites to consider in order to design and process the data share: storage and metadata preparation.

*Storage preparation* a genomic data share often consists of a very large volume of data which can directly affect the cost of data transfer; therefore, the potential cost of the data share point, whether using a local server or cloud storage, must first be evaluated. To estimate the overall cost of the data share, the LDC should collect the following information: the number of samples, the average or maximum size of data per sample, whether or not HIPAA compliant resources are required, sequencing assay types, the list of analysis tools and their licensing terms, effective date of data share, and data governance for data termination and security requirements between data share points. Whenever a local file server is used, at least doubling the size of estimated file storage is recommended for data backup.

*Meta data preparation* in addition to a large data volume, data shares often require a huge list of data elements in various data formats that may need to be updated at any time; therefore, the LDC should be flexible enough to add and change the data models. KCR CRDC uses the end-to-end data share model that can accommodate a wide range of end-user needs and various requirements. This is accomplished by project specific data conversion tools and staging databases that don’t require changing the original data model as shown in Fig. 1d. To maintain the high performance of the end-to-end data model, all data are seamlessly linked together with unique IDs and accessible via programming languages and tools through secure connections while preserving high data accuracy and integrity. Data share is depicted as the one of three categories in the fourth layer of Fig. 4b.

*Data access control* DCs are always challenged by the paradox of maintaining high security while making data broadly accessible. One approach for addressing these two conflicting goals is through role-based access control for the three primary user-roles: admin, developer, and authorized users as shown in Fig. 4c. The admin level in Fig. 4c oversees all databases and manages data integrity and internal and external data transactions. Developers in Fig. 4c are only granted access to specific data points and are responsible for maintaining data integrity and implementing tools for supporting users. Users described as AUTHORIZED USERS in Fig. 4c typically have read-only access to specific cohorts of data as authorized by a data governance committee on a per-project basis. For example, the internal application approval committee in the KCR CRDC determines and grants the level of an applicant’s data access and verifies the status of Institutional Review Board approval. In addition, data access for authorized users depicted as USER in Fig. 4c is facilitated by an honest broker, who either provides deidentified raw data on an isolated computer resource or access to a deidentified user interface like R Shiny Server, LabKey, cBioPortal or Jupyter Notebooks [92].

## Data Availability

Data sharing is not applicable to this article as no datasets were generated or analysed during the current study. The KCR CRDC data harmonization pipeline is freely available at https://github.com/crimcc/bam2vcf. Accessing MCC cBioPortal, LabKey and Tableau molecular data viewer, and on-demand visualization tools needs to contact on crisrf@uky.edu or visit http://crisrf.uky.edu, Cancer Research Informatics Shared Resource Facility (CRI SRF) at Markey Cancer Center.

## Abbreviations

BloodPAC: The Blood Profiling Atlas in Cancer
CRDC: Cancer Research Data Commons
CTDC: Clinical Trial Data Commons
dbGaP: The database of Genotypes and Phenotypes
DC: Data commons
DCC: Data Coordination Center
GDC: Genomic Data Commons
GEO: Gene Expression Omnibus
GUI: Graphic User Interface
HCA: Human Cell Atlas
HIPAA: Health Insurance Portability and Accountability Act
IARC: International Agency for Research on Cancer
ICDS: Integrated Canine Data Commons
ICGC ARGO: International Cancer Genome Consortium Accelerating Research in Genomic Oncology
IDC: Imaging Data Commons
KCR: Kentucky Cancer Registry
LDC: Local Data Commons
MAHE: Manipal Academy of Higher Education
MCC: Markey Cancer Center
MRN: Medical Record Number
MTB: Molecular Tumor Board
NCI: National Cancer Institute
NGS: Next Generation Sequencing
OMOP: Observational Medical Outcomes Partnership
ORIEN: Oncology Research Information Exchange Network
PCA: Principal Component Analysis
PDC: Public Data Commons
QC: Quality control
SBCGC: Seven Bridges Cancer Genomic Cloud
SEER: Surveillance, Epidemiology, and End Results
SSN: Social Security Number
